# Ski mediates TGF-β1-induced fibrosarcoma cell proliferation and promotes tumor growth

**DOI:** 10.7150/jca.46074

**Published:** 2020-08-14

**Authors:** Ping Li, Qiu-Shi Wang, Yu Zhai, Ren-Ping Xiong, Xing Chen, Ping Liu, Yan Peng, Yan Zhao, Ya-Lei Ning, Nan Yang, Yuan-Guo Zhou

**Affiliations:** 1 Department of Army Occupational Disease, State Key Laboratory of Trauma, Burn and Combined Injury, Daping Hospital, Army Medical University (Third Military Medical University), Chongqing 400042, People's Republic of China.; 2 Department of Pathology, Daping Hospital, Army Medical University (Third Military Medical University), Chongqing 400042, People's Republic of China.

**Keywords:** c-Ski, TGF-β1, proliferation, fibrosarcoma, tumor growth

## Abstract

**Background:** TGF-β1 promotes cell proliferation in only some tumors and exerts bidirectional regulatory effects on the proliferation of fibroblasts. This study intends to explore whether the mechanism is related to increased expression of Ski.

**Methods:** Cell proliferation of the fibrosarcoma cell line L929 was assessed with an ELISA BrdU kit. The mRNA and protein expression levels of the corresponding factors were measured by RT-qPCR, immunohistochemistry or Western blotting *in vitro* and *in vivo*. Additionally, c-Ski was knocked down using RNAi. The expression of Ski in human dermatofibrosarcoma protuberans (DFSP) specimens was measured by immunohistochemistry.

**Results:** TGF-β1 promoted the continued proliferation of L929 cells in a dose-dependent manner, with increased c-Ski expression levels. Conversely, inhibition of c-Ski significantly abrogated this unidirectional effect, significantly inhibited the decrease in p21 protein levels and did not affect the increase in p-Smad2/3 levels upon TGF-β1 treatment. Similarly, inhibition of c-Ski significantly abrogated the growth-promoting effect of TGF-β1 on xenograft tumors. Furthermore, we found that high expression of Ski in DFSP was correlated with a low degree of tumor differentiation.

**Conclusions:** Our data reveal that high c-Ski expression is a cause of TGF-β1-promoted proliferation in fibrosarcoma tumor cells and show that inhibiting Ski expression might be effective for treating tumors with high Ski levels.

## Introduction

Transforming growth factor-β1 (TGF-β1), a pivotal cytokine, is involved in the regulation of numerous cellular activities, such as growth, proliferation, differentiation and synthesis of extracellular matrix components 1, 2. Under physiological conditions, TGF-β1 concentrations are 1-10 ng/ml 3, 4, but under pathological conditions, especially in tumors, they can reach 50-60 ng/ml or even higher 5, 6. We previously found that the pro-proliferative effect of a low concentration (0.025 ng/ml) of TGF-β1 is abolished at a high concentration (25 ng/ml) 7. This inhibitory effect at high concentrations is considered critical for the antitumor effect of TGF-β1 8, 9. However, both low and high concentrations of TGF-β1 enhance the proliferation of many tumor cells 10-13 and the growth of tumors and transplanted tumors 14-16. Conversely, inhibition of TGF-β1 or its receptor can decrease tumor cell proliferation and the growth of transplanted tumors 15, 17-19. Although some studies have shown that mutations in TGF-β1 receptors and changes in its downstream signaling pathway are associated with loss of the ability of TGF-β1 to regulate cell proliferation 16, 20, why TGF-β1 always promotes tumor cell proliferation remains unclear.

TGF-β1 performs most of its functions by acting on TGF-β receptors (TGF-βRs) I and II to modulate canonical (Smad-dependent) and noncanonical (Smad-independent) pathways 21, 22. The active TGF-βR complex phosphorylates the receptor-activated Smad proteins (R-Smads) Smad2 and Smad3, and the phosphorylated R-Smads then form heteromeric complexes with a common mediator, Smad4, and translocate into the nucleus through the Smad-dependent pathway 22. In the Smad-independent pathway, TGF-βRs activate other signaling pathways, including the extracellular signal-regulated kinase (ERK), phosphatidylinositol 3-kinase/protein kinase B, and nuclear factor κB pathways 21.

c-Ski, a homolog of the pro-oncogenic v-Ski protein 23 known as Ski in humans, is highly conserved among different species. c-Ski, a versatile transcriptional regulator, has been reported to negatively regulate the TGF-β/Smad signaling pathway through interactions with Smad2, Smad3 and Smad4 24, 25. Moreover, c-Ski regulates other signaling pathways by promoting the transcription of nuclear factor I, forming complexes with the retinoic acid receptor (RAR) and inhibiting the Rb-mediated signaling pathway 26-28. TGF-β1 can bypass the effects of the Smad2/3 signaling pathway on the E3 ubiquitin ligase Arkadia to mediate rapid c-Ski degradation 29. In addition, c-Ski plays an essential role in the TGF-β1-mediated bidirectional regulation of primary fibroblast cell proliferation, as c-Ski expression is increased at low TGF-β1 concentrations and reduced at high TGF-β1 concentrations 7, 30. In addition, TGF-β1 is highly expressed in most human tumors 31-34. Determining whether Ski expression is also high in these tumors and determining whether Ski mediates TGF-β1 function will provide new mechanistic insight into TGF-β1-promoted tumor cell proliferation.

To test our hypothesis, we used mesenchymal stem cell-derived fibroblasts and fibrosarcoma cells (L929) for this study, which was based on our previous research results. In addition, we used *c-Ski* RNA interference (RNAi) combined with TGF-β1 stimulation to demonstrate that the pro-proliferative effect of TGF-β1 depends on high c-Ski expression. Moreover, we verified the above results *in vivo* using a fibrosarcoma xenograft tumor model. In addition, we investigated whether the mechanism by which c-Ski promotes proliferation is related to the inhibition of Smad2/3 and p21. Finally, we used human dermatofibrosarcoma protuberans (DFSP) specimens, which have high TGF-β1 expression, to confirm the correlation between Ski expression and the degree of tumor differentiation. These findings clarify the role of Ski in TGF-β1-promoted tumor cell proliferation and highlight Ski as a detectable therapeutic target for cancer.

## Materials and Methods

### Antibodies and reagents

Antibodies specific for Smad3 (#9523), p-Smad3 (Ser423/425, #9520), Smad2 (#3103), and p-Smad2 (Ser465/467, #3101) were purchased from Cell Signaling Technology (Danvers, MA, USA). Antibodies specific for p-Smad2/3 (sc-11769), Ski (sc-9140), p21 (sc-397), and actin (sc-1616) were purchased from Santa Cruz Biotechnology (Dallas, TX, USA). HRP-conjugated anti-rabbit (specific for Smad3, p-Smad3, p-Smad2, Ski, p21, and actin) or anti-mouse (specific for Smad2) antibodies were purchased from Santa Cruz Biotechnology (Dallas, TX, USA). Anti-rabbit FITC (ZF-0311), anti-mouse TRITC (ZF-0313), and anti-goat TRITC (ZF-0317) were purchased from ZhongShan Golden Bridge Biotechnology (Beijing, China).

### Cell lines and mice

The L929 mouse fibrosarcoma cell line was acquired from the American Type Culture Collection (ATCC CCL-1™, Rockville, MD). Primary fibroblast cultures were established as published previously 7, and fibroblasts at passages 3-8 were used for the experiments. Adult female BALB/c-nu/nu nude mice, 4-6 weeks old and weighing 18-20 g, were provided by the Animal Center of Daping Hospital, Third Military Medical University (Certificate scxk (Yu) 2002-0002, Chongqing, China). The experimental procedures were performed in accordance with the guidelines of the Animal Ethical and Welfare Committee of the Third Military Medical University.

### Construction of a c-Ski siRNA-expressing plasmid

The pSuper-c-Ski-RNAi and pSuper-control RNAi plasmids were constructed in our previous study 7 with the following oligonucleotide sequences: c-Ski, 5′-GATCCCCGAAGGAGTTGGCGGCCAGCTTCAAGAGAGCTGGCCGCCAACTCCTTCTTTTTA-3′ (sense) and 5′-AGCTTAAAAAGAAGGAGTTGGCGGCCAGCTCTCTTGAAGCTGGCCGCCAACTCCTTCGGG-3′ (antisense); control RNAi, 5′-GATCCCCGACTTCATAAGGCGCATGCTTCAAGAGAGCATGCGCCTTATGAAGTCTTTTTA-3′ (sense) and 5′-AGCTTAAAAAGACTTCATAAGGCGCATGCTCTCTTGAAGCATGCGCCTTATGAAGTCGGG-3′ (antisense).

### Detection of cell proliferation and transient transfection

A total of 1×10^4^ cells were plated into 96-well plates and incubated overnight. At 24 h after synchronization with FBS-free DMEM, the cells were left untreated or were treated with TGF-β1 for the indicated times at doses ranging from 0.0025 ng/ml to 250 ng/ml. Cell proliferation was assessed as previously described using a BrdU ELISA kit (Roche, Indianapolis, IN, USA).

For transfection experiments, cells were treated with DMEM (control) or transfected with the pSuper-c-Ski-RNAi or pSuper-control RNAi plasmid using Lipofectamine 2000 (Invitrogen, Carlsbad, CA, USA). TGF-β1 was added 24 h after transfection, and proliferation was detected as described above after an additional 24 h.

### Quantitative real-time PCR (RT-PCR) and Western blotting

At 24 h after synchronization with FBS-free DMEM, untransfected and transfected cells were treated with or without TGF-β1 (0.25 ng/ml or 25 ng/ml) for 4 h or 24 h. RNA was then extracted using TRIzol® reagent (Invitrogen, Carlsbad, CA, USA) according to the manufacturer's protocol, and total cell lysates were harvested for Western blotting, which was performed as described in our previous publication 7. The relative quantity of each target protein was normalized to that of actin. In addition, SYBR Green I-based quantitative real-time PCR was performed as previously described 32. The RT-PCR primer pairs were as follows: Smad3, 5′-TCCTGGCTACCTGAGTGAAGA-3′ (sense) and 5′-GTTGGGAGACTGGACGAAAA-3′ (antisense); c-Ski, 5′-TCAACTCCGGTGTGCGATG-3′ (sense) and 5′-CGTCCGTCTTAGTGATGAG-3′ (antisense); and glyceraldehyde-3-phosphate dehydrogenase (GAPDH), 5′-AGGTTGTCTCCTGCGACTTCA-3′ (sense) and 5′-TGGTCCAGGGTTTCTTACTCC-3′ (antisense). The thermocycler program was set as follows: 5 min at 94°C, followed by 40 cycles of 15 s at 94°C, 15 s at 59°C and 20 s at 72°C. The relative expression levels of genes were calculated by the 2-ΔΔCt method 35 and normalized to those of the housekeeping gene GAPDH.

### Immunofluorescence staining

The transfected cells were pretreated with or without TGF-β1 for 4 h or 24 h and then subjected to immunofluorescence analysis as previously described 27, 36. After incubation with the anti-p-Smad2/3, anti-c-Ski, anti-c-Ski or anti-p21 antibodies (1:100) overnight at 4°C, the sections were labeled with a FITC- or TRITC-conjugated secondary antibody (1:200) for 1 h at 37°C. Finally, the slides were stained with DAPI for 15 min at 37°C and then observed under a fluorescence microscope (Leica, Wetzlar, Germany). For the controls, the primary antibody was replaced with normal rabbit or goat immunoglobulin (control Ab).

### Cancer models and tumor quantification *in vivo*

Mice aged 5 weeks received subcutaneous injections of 5×10^6^ cells in 200 μl of PBS in the armpit of the left forelimb and were returned to their cages for 2 weeks until xenograft tumors formed. Then, the mice were randomly divided into four groups of nine mice each that were treated as follows (3 animals per cage): (1) the TGF-β1 treatment group received an intratumoral injection of 100 ng of TGF-β1 in 50 μl of PBS; (2) the PBS control group received an intratumoral injection of 50 μl of PBS alone; (3) the c-Ski-RNAi group received an intratumoral injection of 100 μg of pSuper-c-Ski-RNAi plasmid in 50 μl of PBS 36, 37; and (4) the pSuper-control RNAi group received an intratumoral injection of 100 μg of pSuper-control RNAi plasmid in 50 μl of PBS. All of the groups received injections twice per week for a total of 1.0 weeks. All animals were housed as previously described 38.

All animals were evaluated twice weekly until the end of the experiment to monitor body weight gain and tumor growth. The tumor volume was approximated using the following equation: volume=(a×b^2^)/2 39, where a is the length of the major axis and b is the length of the minor axis.

### Immunofluorescence analysis of xenograft tumors

Three and a half weeks after grafting, the mice were sacrificed by cervical dislocation. The tumors were removed, fixed in formaldehyde solution and embedded in paraffin according to a standard protocol. Hematoxylin and eosin (H&E) staining was performed as described in our previous publication 40, and immunofluorescence analysis was performed as previously described 41. Primary antibodies against p-Smad2/3 and c-Ski (1:100) and the corresponding FITC- and TRITC-conjugated secondary antibodies (1:200) were used. For the controls, the primary antibody was replaced with normal rabbit immunoglobulin. Fluorescence was visualized under a fluorescence microscope. The immunofluorescence data were analyzed as previously described using the integrated optical density 42, 43.

### Clinical data and histology

Specimens from twenty-five cases of DFSP were obtained from Daping Hospital and Xinan Hospital of the Third Military Medical University (Chongqing, China), excluding patients who had previously been treated with chemotherapy or radiation therapy and patients who lacked clinical data. The demographic and clinical data of the subjects, including age, sex, tumor site, tumor size (mm), tumor type (primary or recurrent), and tumor differentiation grade (well-differentiated, moderately differentiated, poorly differentiated or undifferentiated), were obtained from patient records.

Immunohistochemical staining was performed according to a standard protocol using 5-µm sections. Samples incubated with a primary antibody (c-Ski, 1:100) and negative control samples were examined. The staining intensity of the cells was scored on a 4-category scale ranging from 0-3 as follows: no staining, 0; weak staining, 1+ (light brown staining); intermediate staining, 2+; and strong staining, 3+ (dark brown staining). Quantitative analyses were performed using at least six different sections, and staining was scored independently by two observers who were blinded to the treatment conditions; the average score is presented.

### Statistical analysis

The data were analyzed using the SPSS statistical software package (standard version 18.0; SPSS, Chicago, USA). The associations of Ski expression, age, and sex with the DFSP tumor differentiation grade were assessed by multivariate logistic regression. Comparisons of the other *in vitro* and *in vivo* data were performed with Student's t-test, and the values are expressed as the mean ± SE.

## Results

### c-Ski expression mediates TGF-β1-induced bidirectional effects on mouse fibroblast proliferation

Consistent with previous reports 7, TGF-β1 exhibited bidirectional effects on the proliferation of fibroblasts; e.g., low concentrations of TGF-β1 (0.0025 ng/ml to 2.5 ng/ml, *P<*0.01) stimulated cell proliferation, whereas high concentrations (25 ng/ml and 250 ng/ml, *P<*0.01, Figure S1A) inhibited cell proliferation. Similarly, low concentrations of TGF-β1 (0.25 ng/ml) significantly increased the expression of c-Ski mRNA, which was significantly inhibited at high concentrations of TGF-β1 (25 ng/ml, Figure S1B). Moreover, the bidirectional effects were eliminated after c-Ski overexpression by plasmid transfection or inhibition by RNAi (Figure S1C-D).

### Effect of c-Ski on the unidirectional regulation of L929 cell proliferation by TGF-β1

**Figure 1:**
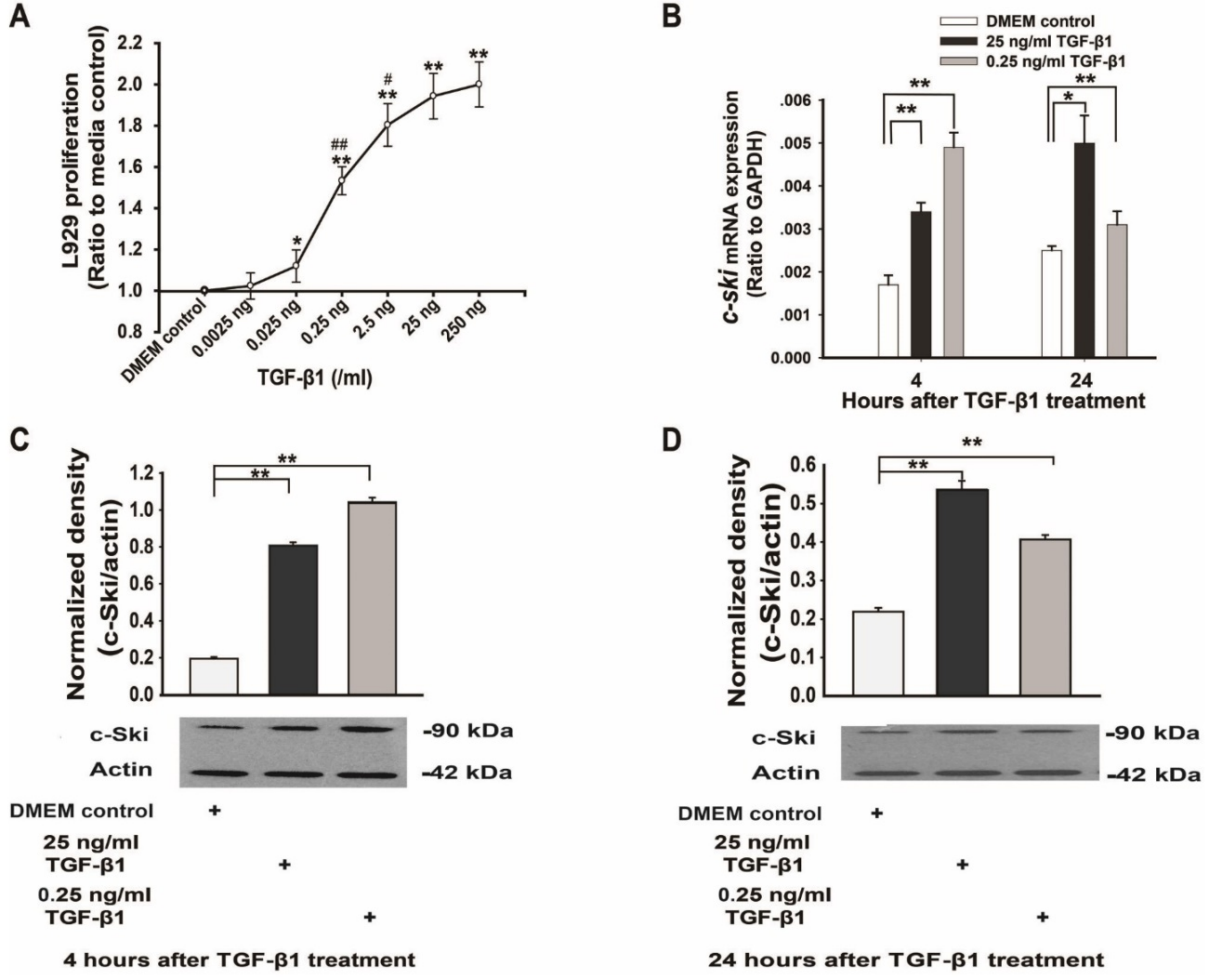
** Changes in L929 cell proliferation and Ski expression levels after TGF-β1 treatment. A.** TGF-β1 increased L929 cell proliferation in a dose-dependent manner (n=12), as measured with an ELISA kit (Roche); Student's t-test, *P<0.05, **P<0.01 vs. the control; #*P*<0.05, ##*P*<0.01 vs. the adjacent dose. **B.** Changes in *c-Ski* mRNA expression at 4 h and 24 h after TGF-β1 treatment (n=9); Student's t-test, *P<0.05, **P<0.01 vs. the control. Changes in c-Ski protein expression at 4 h (**C**) and 24 h (**D**) after TGF-β1 treatment (n=3) in three independent experiments; Student's t-test, **P<0.01 vs. the control.

**Figure 2:**
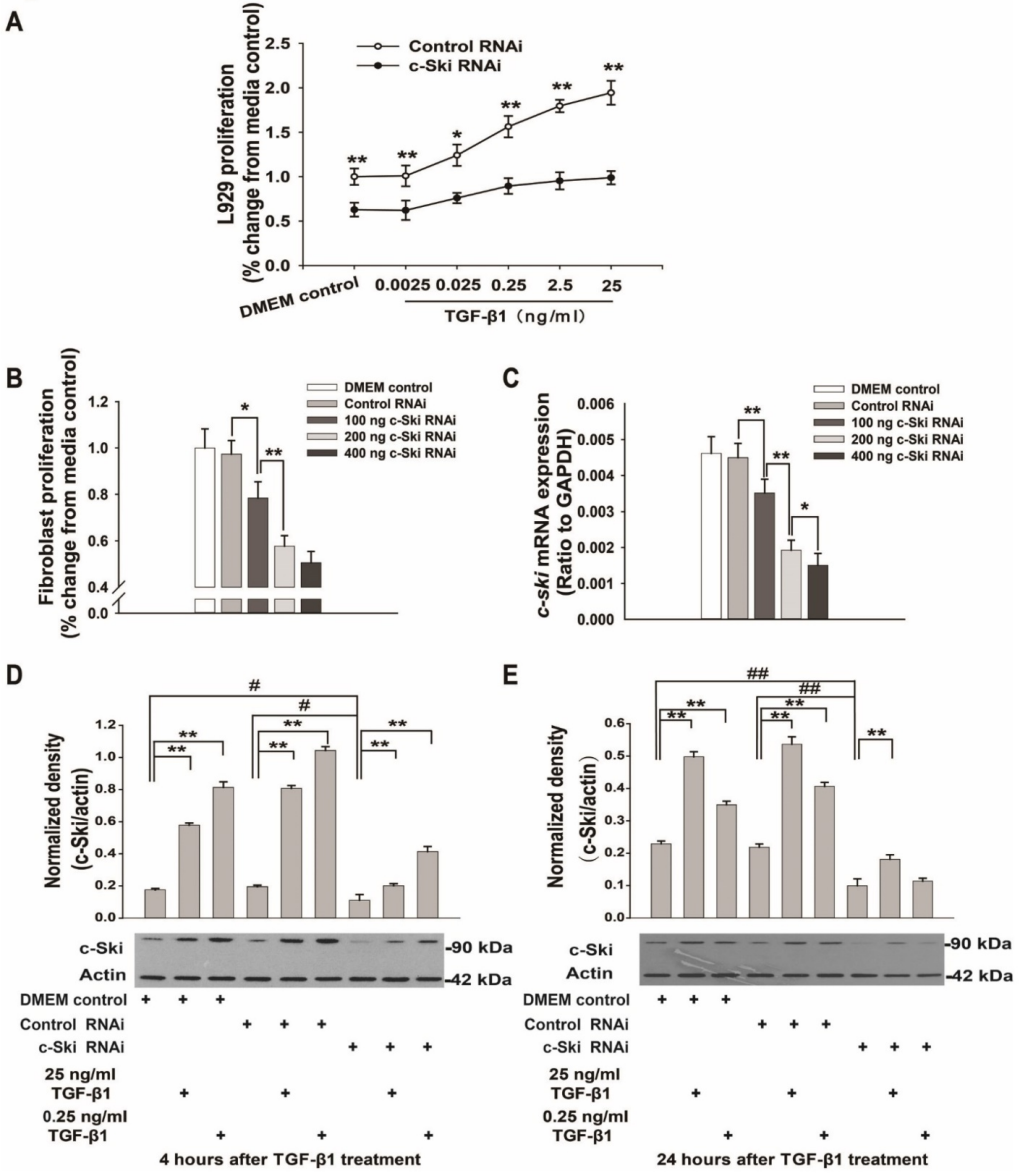
** Effects of TGF-β1 on L929 cell proliferation after transfection with 200 ng of c-Ski-RNAi plasmid. A.** c-Ski knockdown significantly inhibited the proliferative effects of different concentrations of TGF-β1 (n=12); Student's t-test, *P<0.05, **P<0.01 vs. the corresponding dose of control RNAi. **B.** Effects of different doses of the c-Ski-RNAi plasmid on proliferation (n=12); Student's t-test, *P<0.05, **P<0.01 vs. the adjacent dose. **C.** Changes in *c-Ski* mRNA expression after injection of different doses of the c-Ski-RNAi plasmid (n=9); Student's t-test, *P<0.05, **P<0.01 vs. the adjacent dose. Changes in c-Ski protein expression following TGF-β1 treatment for 4 h (**D**) and 24 h (**E**) after injection of 200 ng of c-Ski RNAi (n=3) in three independent experiments; Student's t-test, **P<0.01 vs. the control in each group; #*P<*0.05, ##*P<*0.01 vs. control RNAi.

To verify whether TGF-β1 exerts bidirectional effects on L929 cell proliferation and to ascertain whether the role of c-Ski in this process is similar to its previously identified role in fibroblasts 7, L929 cells were treated with different concentrations of TGF-β1 for 24 h. The data demonstrated that TGF-β1 promoted L929 cell proliferation in a dose-dependent manner (0.0025 ng/ml to 250 ng/ml, *P<*0.05, Figure 1A) and that c-Ski mRNA and protein levels were significantly increased by 25 ng/ml and 0.25 ng/ml TGF-β1 (Figure 1B-D). In contrast, cells transfected with c-Ski-RNAi showed significant dose-dependent decreases in c-Ski mRNA expression levels (Figure 2C) and c-Ski protein levels (Figure 2D and E), accompanied by significant decreases in cell proliferation (Figure 2B); in addition, the TGF-β1-mediated increases in c-Ski protein levels were abolished in these cells (Figure 2D and E), as was the unidirectional effect of TGF-β1 on L929 cell proliferation (Figure 2A). These results indicated that c-Ski is a key determinant of the unidirectional effects of TGF-β1 on L929 cell proliferation.

### Effects of c-Ski on Smad2/3 and p21 regulation

**Figure 3:**
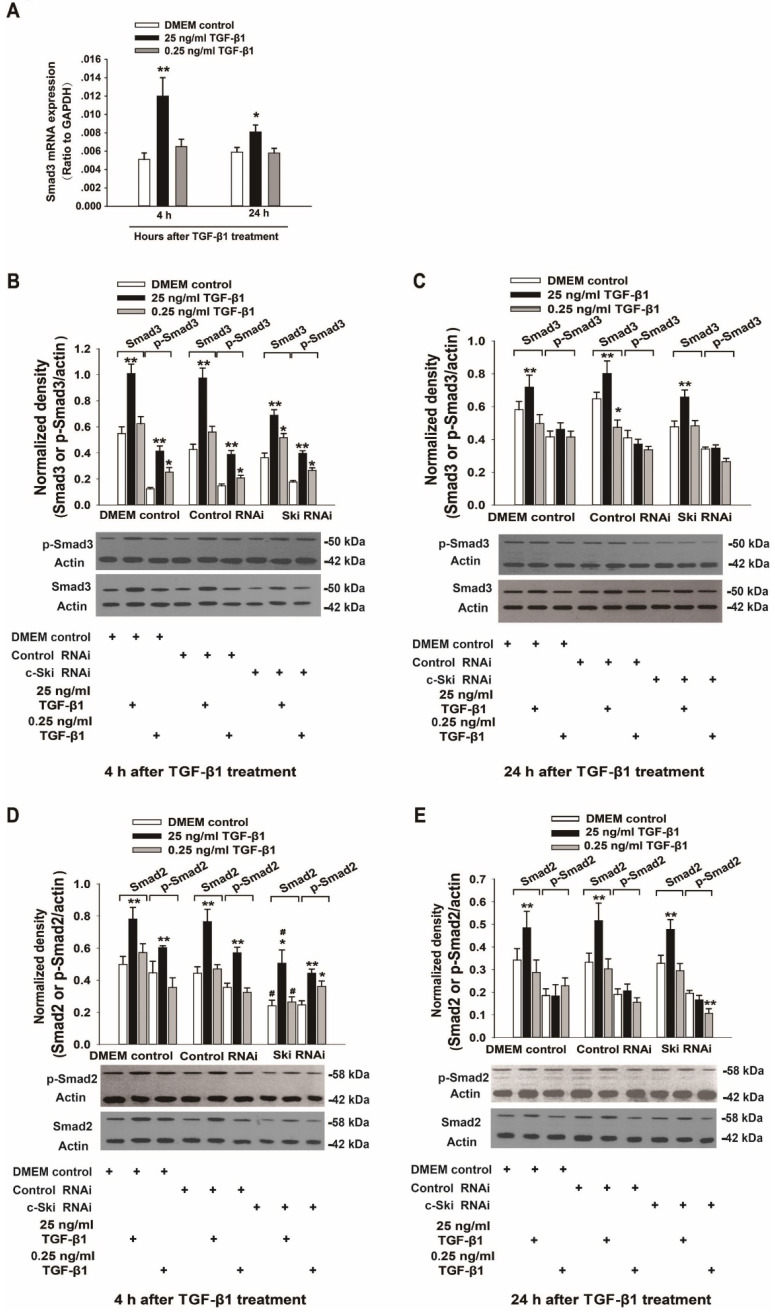
** Changes in the expression and activity of Smad2/3 upon regulation of c-Ski expression levels. A.** Changes in *Smad3* mRNA expression at 4 h and 24 h after TGF-β1 treatment (n=9); Student's t-test, *P<0.05, **P<0.01 vs. the control. Changes in Smad3 and Smad2 protein and phosphorylation levels following TGF-β1 treatment for 4 h (**B, D**) and 24 h (**C, E**) after transfection with c-Ski RNAi (n=3) in three independent experiments; Student's t-test, *P<0.05, **P<0.01 vs. the control in each group; #*P<*0.05, ##*P<*0.01 vs. control RNAi.

**Figure 4:**
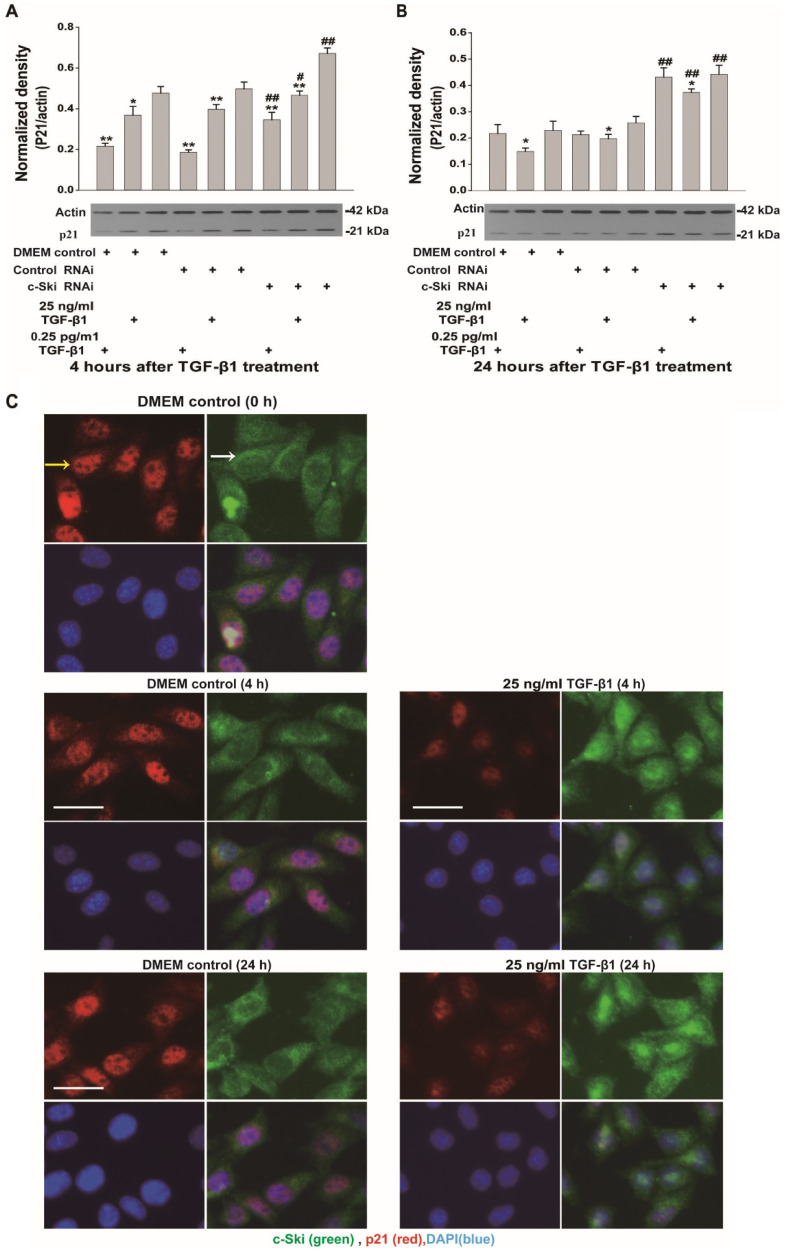
** Changes in p21 expression upon regulation of c-Ski expression levels in L929 cells.** Changes in p21 and Smad2 protein expression after treatment with TGF-β1 for 4 h (**A**) and 24 h (**B**) after transfection with c-Ski RNAi (n=3) in three independent experiments; Student's t-test, *P<0.05, **P<0.01 vs. the control in each group; #*P<*0.05, ##*P<*0.01 vs. control RNAi. **C,** Immunofluorescence analysis of c-Ski (green, white arrow) and p21 (red, yellow arrow) at 4 h and 24 h after treatment with the high dose of TGF-β1 (25 ng/ml). Nuclei are indicated by DAPI staining (blue); scale bar=20 µm.

Because c-Ski acts as a corepressor of Smad3 transcription, we also investigated the expression and activation of Smad3 and p21 (a transcription factor downstream of Smad3) upon the induction of L929 cell proliferation by TGF-β1. The mRNA, protein and phosphorylation levels of Smad3 in L929 cells significantly increased after stimulation with 25 ng/ml TGF-β1 and did not significantly change upon treatment with 0.25 ng/ml TGF-β1 (Figure 3A-C); these changes were not significantly affected by RNAi-mediated inhibition of c-Ski expression (Figure 3B and C). In addition, the changes in Smad2 protein and phosphorylation levels were similar to the changes in Smad3 protein and phosphorylation levels (Figure 3D and E). Fluorescence immunocytochemistry showed that TGF-β1 increased p-Smad2/3 levels in the nucleus and that the fluorescence intensity of c-Ski increased upon treatment with 25 ng/ml TGF-β1 (Figure S2). Furthermore, the decreases in p21 protein levels were significantly increased after RNAi-mediated inhibition of c-Ski expression and 4 h of TGF-β1 stimulation and were restored to the levels in untreated cells at 24 h after TGF-β1 treatment (Figure 4A and B). In addition, immunocytochemistry showed that 25 ng/ml TGF-β1 increased the fluorescence intensity of c-Ski and decreased that of p21 (Figure 4C). These experiments showed that the increase in tumor cell proliferation caused by high c-Ski expression is associated with inhibition of P21 expression and the Smad-independent pathway.

### Effects of c-Ski expression on TGF-β1-mediated L929 tumor growth in a xenograft mouse model

**Figure 5:**
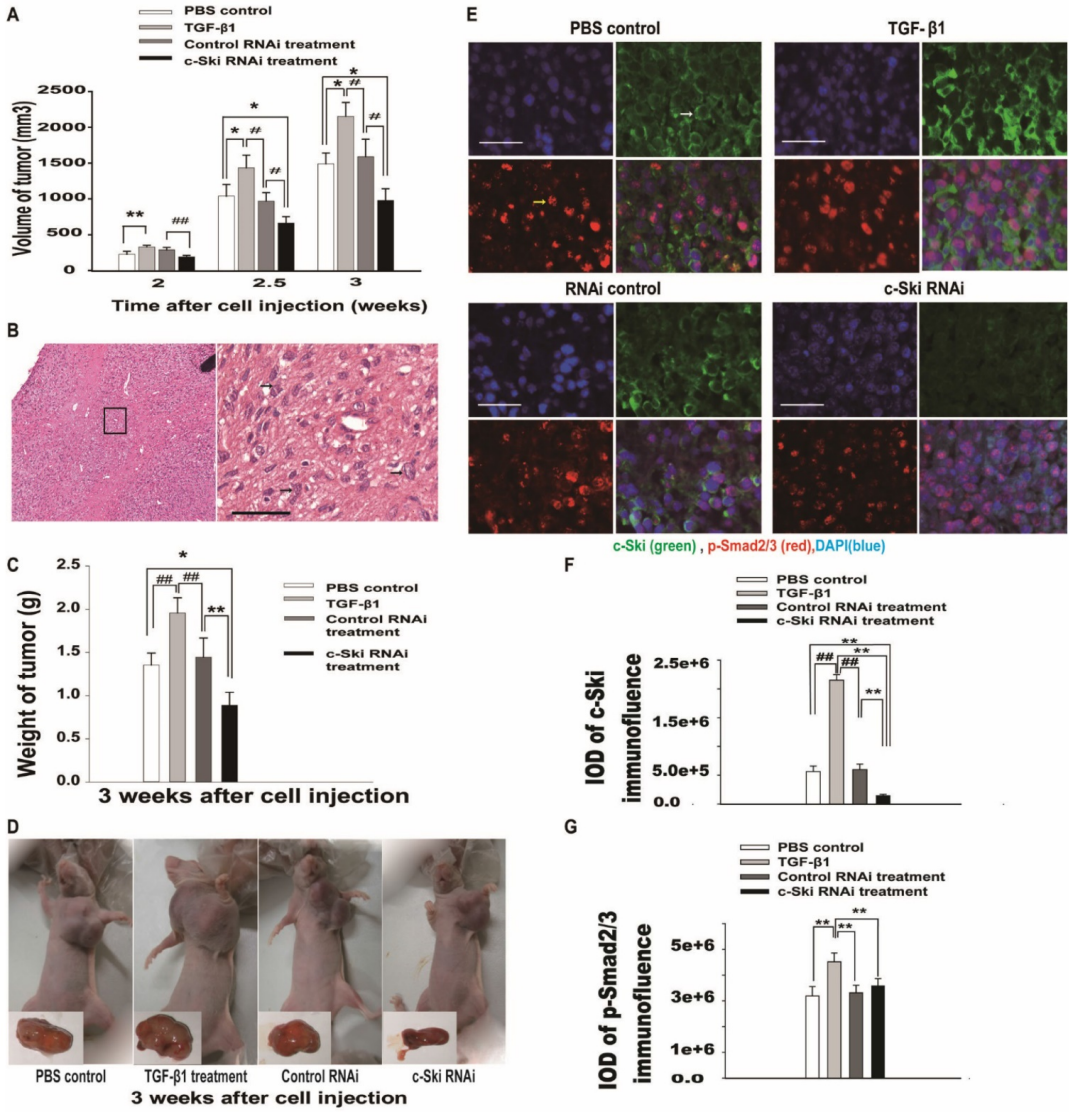
** Effects of 100 ng of TGF-β1 and/or 100 µg of c-Ski-RNAi plasmid on L929 xenograft tumor growth *in vivo*. A.** Tumor growth curves for the different groups 2-3.5 weeks after subcutaneous injection (n=9); Student's t-test, *P<0.05, **P<0.01 vs. c-Ski RNAi; #*P<*0.05, ##*P<*0.01 vs. TGF-β1 treatment. **B.** Histologically, the xenograft tumors featured higher cellularity and more pronounced cell atypia (black arrow) than normal tissue at 2 weeks after injection; left panels, large images, scale bar = 20 µm. **C.** Tumor weights in the different groups at 3.0 weeks after injection (n=9); Student's t-test, *P<0.05, **P<0.01 vs. c-Ski RNAi; #*P<*0.05, ##*P<*0.01 vs. TGF-β1 treatment. **D.** Photographs of xenograft tumors in different groups of mice at 3.0 weeks after injection. Lower right, photographs of excised xenografts. Scale bars=200 µm (small picture), 50 µm (large picture). **E.** Immunofluorescence showing the levels of c-Ski (green, white arrow) and p-Smad2/3 (red, yellow arrow) in xenograft tumors at 3.0 weeks after injection. Nuclei are indicated by DAPI staining (blue); scale bar=50 µm. Relative expression levels of c-Ski (**F**) and p-Smad2/3 (**G**) in xenograft tumors; Student's t-test, *P<0.05, **P<0.01 vs. c-Ski RNAi; #*P<*0.05, ##*P<*0.01 vs. TGF-β1 treatment.

To investigate the effects of c-Ski on tumor growth mediated by TGF-β1 *in vivo*, we used a mouse xenograft tumor model. Two weeks after subcutaneous injection of 5×10^6^ cells into the armpit of the left forelimb, each mouse was treated with PBS, TGF-β1, pSuper-c-Ski-RNAi plasmid or pSuper-control RNAi plasmid. The results showed that the axillary tumors had developed to volumes of approximately 234 ± 54 mm^3^ at 2 weeks in PBS control mice (Figure 5A). Moreover, the higher cellularity and more pronounced cell atypia visualized by H&E staining (Figure 5B) revealed the existence of poorly differentiated tumors in the control group. Compared with the PBS control, 100 ng of TGF-β1 significantly promoted tumor growth (Figure 5A, C and D). However, compared with the control RNAi group, the control siRNA group showed no significant change, and the c-Ski RNAi group showed decreases of 53.8% in tumor volume and 48.8% in weight (Figure 5A and C) 3.0 weeks after tumor cell injection.

Fluorescence immunohistochemistry showed that c-Ski protein levels increased after treatment with TGF-β, decreased after exposure to c-Ski RNAi and were not changed significantly by exposure to control siRNA (Figure 5E and Figure S3). Moreover, p-Smad2/3 levels were increased in the TGF-β1 treatment group compared to the control group, while the other groups showed no significant differences (Figure 5F and G). These results suggested that c-Ski may play a role in TGF-β1-mediated L929 tumor growth *in vivo*.

### Ski expression in mesenchymal stem cell-derived DFSP and sarcomas

**Figure 6:**
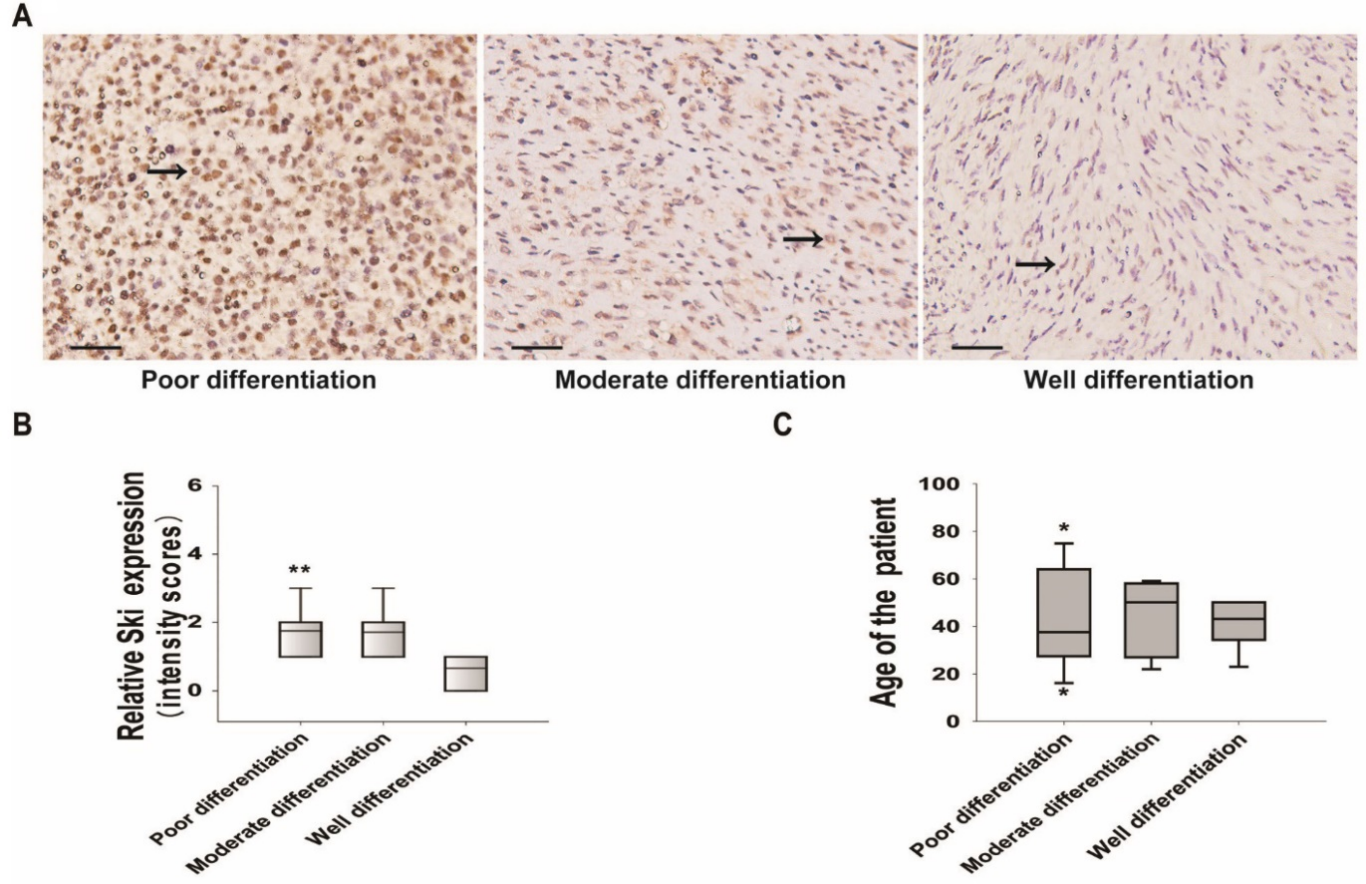
** Changes in Ski expression and correlations between Ski expression and DFSP characteristics. A.** Immunohistochemical analysis of Ski in DFSP specimens with different degrees of differentiation (well-differentiated, n=7; moderately differentiated, n=7; poorly differentiated, n=11). Positive Ski expression appears as pale brown staining (black arrow) in the cytoplasm and nucleus; scale bars=50 µm. **B.** Correlations between Ski expression levels and the differentiation of DFSP; multivariate logistic regression, **P<0.01 vs. the well-differentiated group. **C.** Correlations between Ski expression levels and the average age of DFSP patients in different groups.

We collected specimens from twenty-five cases of human DFSP with high TGF-β1 expression and investigated Ski expression. Of the 25 patients who provided specimens, 60% were men and 40% were women, and the mean age was 43 ± 16.3 years (Table SI). The positive rate of Ski expression was 92%. Ski expression was low in well-differentiated sarcomas but high in moderately and poorly differentiated sarcomas (Figure 6A and B). Although only 25 cases were included, multinomial logistic regression analysis showed that Ski expression was related to the degree of sarcoma differentiation (Table SII). In particular, Ski expression in well-differentiated sarcomas was significantly different from that in poorly differentiated sarcomas (Figure 6B and Table SII). Age (Figure 6C and Table SII) and sex (Table SII) were not related to sarcoma differentiation. These results showed that high c-Ski levels correlate with the degree of DFSP differentiation.

## Discussion

### Unidirectional promotion of murine fibrosarcoma L929 cell proliferation by TGF-β1 in a dose-dependent manner

In contrast to a previous finding that TGF-β1 exerts bidirectional regulatory effects on the proliferation of rat fibroblasts 7, we found similar results in primary fibroblasts from mice. More importantly, we also found that TGF-β1 promoted the proliferation of murine fibrosarcoma L929 cells in a dose-dependent manner over a range of 0.0025 pg/ml to 25 ng/ml. Although we previously confirmed 44 that the peak TGF-β1 concentration in the supernatant is significantly higher (approximately 10-fold higher) for L929 cells *in vitro* than for fibroblasts *in vitro*, 25 ng/ml exogenous TGF-β1 still promoted L929 cell proliferation, indicating that TGF-β1 can induce strong and stable tumor cell proliferation. In addition, a growing number of recent studies have shown that TGF-β1 may not inhibit but rather promote the proliferation of many types of cancer cells 31, 45. These findings suggest that TGF-β1 has pro-proliferative effects on a wide range of tumors, a possibility that should not be ignored.

### Elevated c-Ski expression is a key factor in the unidirectional pro-proliferative effects of high concentrations of TGF-β1 on L929 cells

In the current study, we found that TGF-β1 mediated bidirectional regulation of mouse-derived fibroblast proliferation by increasing or decreasing c-Ski expression (Figure S2), which is consistent with the findings of our previous study 7. In contrast, TGF-β1 induced the proliferation of L929 cells at all concentrations and elevated c-Ski expression in a dose-dependent manner (Figure 1A); moreover, the effect of 0.25 ng/ml TGF-β1 was stronger but shorter than that of 25 ng/ml TGF-β1. Moreover, the unidirectional effect was lost after c-Ski expression was knocked down by RNAi (Figure 2A). Therefore, high c-Ski expression is an important factor in the unidirectional pro-proliferative effect of TGF-β1 in L929 cells. The current study also showed that TGF-β1 significantly promoted the growth of mouse xenograft tumors (Figure 5A and C), which had significantly elevated c-Ski expression (Figure 5E). Moreover, interfering with c-Ski expression significantly inhibited xenograft tumor growth (Figure 5A and C). Taken together, these results further confirm the important role of c-Ski in the pro-proliferative effects of TGF-β1 on L929 cells. Notably, interfering with c-Ski expression did not enable the same bidirectional regulation of proliferation by TGF-β1 observed during fibrosis, perhaps due to incomplete c-Ski knockdown by RNAi or for other reasons.

It has been reported that TGF-β1 can decrease Ski levels through the E3 ubiquitin ligase Arkadia in the Smad-dependent pathway, but in the current study, high c-Ski expression was promoted rather than inhibited by TGF-β1 in L929 cells, indicating that TGF-β1 can also regulate Ski levels in other ways. As we recently discovered, low TGF-β1 concentrations induce c-Ski expression through the ERK/CREB pathway during primary fibroblast proliferation 46. However, the ERK/CREB pathway and other pathways that regulate Ski levels require further study.

### Increased c-Ski expression stimulated by high concentrations of TGF-β1 may be related to decreased p21 expression

It has been reported that c-Ski promotes tumor cell proliferation by inhibiting p21 expression induced by the TGF-β1/Smad pathway 47-49. We found that the change in c-Ski expression induced by TGF-β1 was consistent with the change in p21 expression (Figure 4), which increased significantly after c-Ski knockdown. This result indicates that regulation of p21 by high c-Ski expression is an important mechanism for the unidirectional pro-proliferative effect of TGF-β1 in L929 cells. In addition, increases in Smad2/3 expression and phosphorylation levels were observed in L929 cells after treatment with different doses of TGF-β1, different from the inhibition observed in fibroblasts after treatment with low doses of TGF-β1 7. Moreover, Smad2/3 expression and phosphorylation levels in mouse xenograft tumors did not change with c-Ski overexpression or knockdown (Figures 3 and 5). Taken together, these results suggest that the decrease in p21 does not occur through the Smad2/3 pathway but instead through a Smad-independent pathway that is more likely to inhibit p21 transcriptional activity. Similarly, Ijichi et al. 50 demonstrated Smad-independent regulation of p21 in tumors.

### Ski may be a diagnostic marker and therapeutic target

Although c-Ski expression was low in normal fibroblasts and although c-Ski knockdown did not affect fibroblast proliferation 7, Ski has been shown to be highly expressed in many human malignant tumors 51, and downregulation of Ski decreases pancreatic tumor growth 51. We demonstrated for the first time that Ski was expressed in 92% of human DFSP samples of mesenchymal origin. Furthermore, although the number of cases was limited, a negative correlation between Ski expression and the degree of tumor differentiation was observed in our study. More importantly, RNAi-mediated downregulation of c-Ski significantly reduced L929 cell proliferation in a dose-dependent manner and abrogated xenograft tumor growth in mice. Thus, the above findings all suggest that Ski may be a good marker of tumor differentiation in terms of tumor cell proliferation or malignancy and may be a target for the treatment of tumors.

Our results first demonstrated that increased c-Ski expression is critical for the unidirectional pro-proliferative effect of TGF-β1 on fibrosarcoma cells and *in vivo* tumors. Additionally, our findings showed that high Ski levels may be related to high TGF-β1 levels. Furthermore, c-Ski downregulation inhibited tumor cell proliferation *in vitro* and tumor growth *in vivo* in this study. More importantly, Ski expression was observed in human DFSP samples, which showed high TGF-β1 expression, and was related to the degree of tumor differentiation. These findings hold promise for future clinical applications related to treating tumors and diagnosing the degree of malignancy.

## Supplementary Material

Supplementary figures and tables.
