# Supplementary material for: Ski mediates TGF-β1-induced fibrosarcoma cell proliferation and promotes tumor growth
Source: J Cancer. 2020 Aug 14;11(20):5929–40. doi: 10.7150/jca.46074 (PMC7477421; doi:10.7150/jca.46074)
Supplement: Supplementary file 1 — Supplementary figures and tables. [file jcav11p5929s1.pdf]

Fig. S1

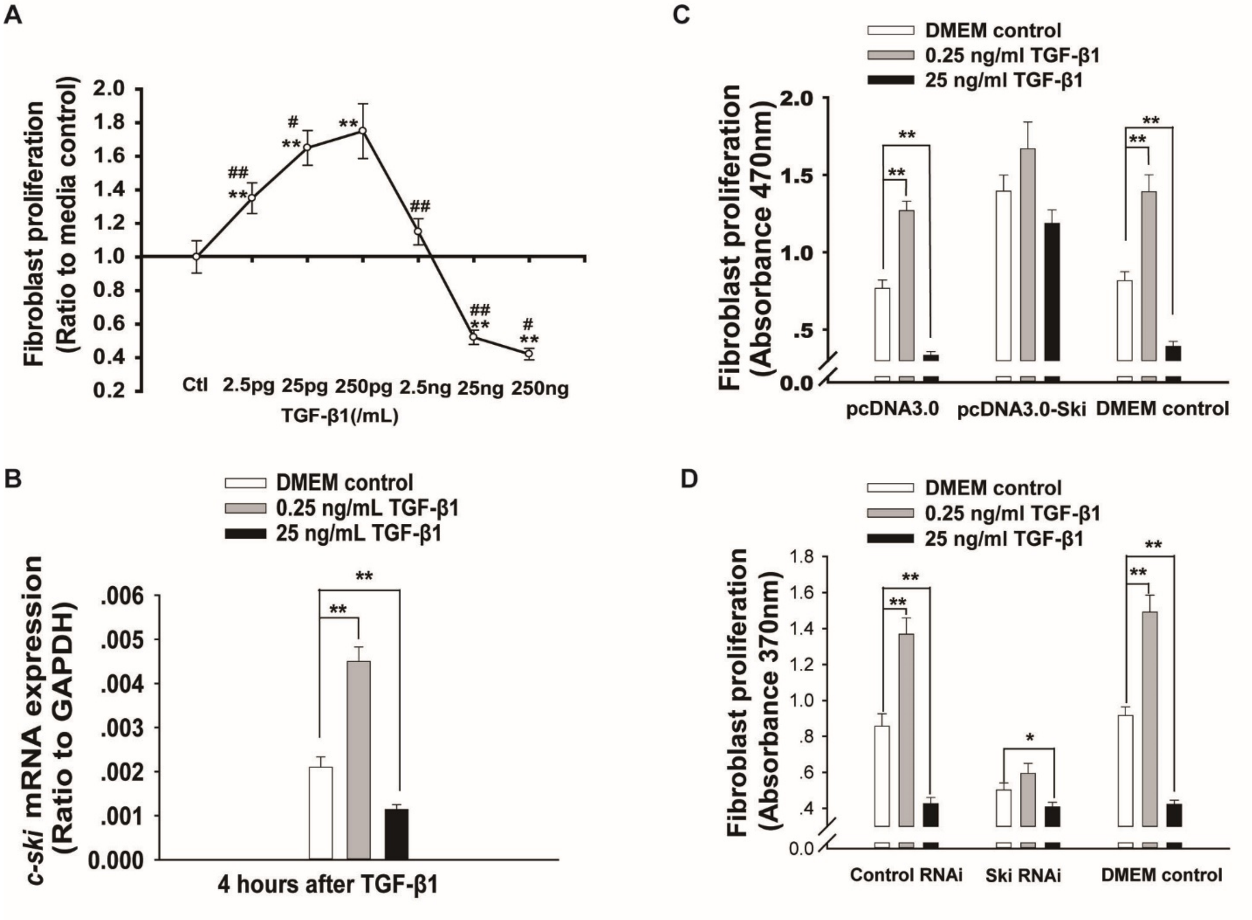
A
Fibroblast proliferation (Ratio to media control)
2.0
1.8
1.6
1.4
1.2
1.0
0.8
0.6
0.4
0.2
##
**
#
**
**
##
##
**
#
**
Ctl 2.5pg 25pg 250pg 2.5ng 25ng 250ng
TGF-β1(/mL)
B
DMEM control
0.25 ng/mL TGF-β1
25 ng/mL TGF-β1
c-ski mRNA expression (Ratio to GAPDH)
.006
.005
.004
.003
.002
.001
0.000
**
**
4 hours after TGF-β1
C
DMEM control
0.25 ng/ml TGF-β1
25 ng/ml TGF-β1
Fibroblast proliferation (Absorbance 470nm)
2.0
1.5
1.0
.5
0.0
**
**
**
**
pcDNA3.0 pcDNA3.0-Ski DMEM control
D
DMEM control
0.25 ng/ml TGF-β1
25 ng/ml TGF-β1
Fibroblast proliferation (Absorbance 370nm)
1.8
1.6
1.4
1.2
1.0
.8
.6
.4
0.0
**
**
*
**
**
Control RNAi Ski RNAi DMEM control

Fig. S2

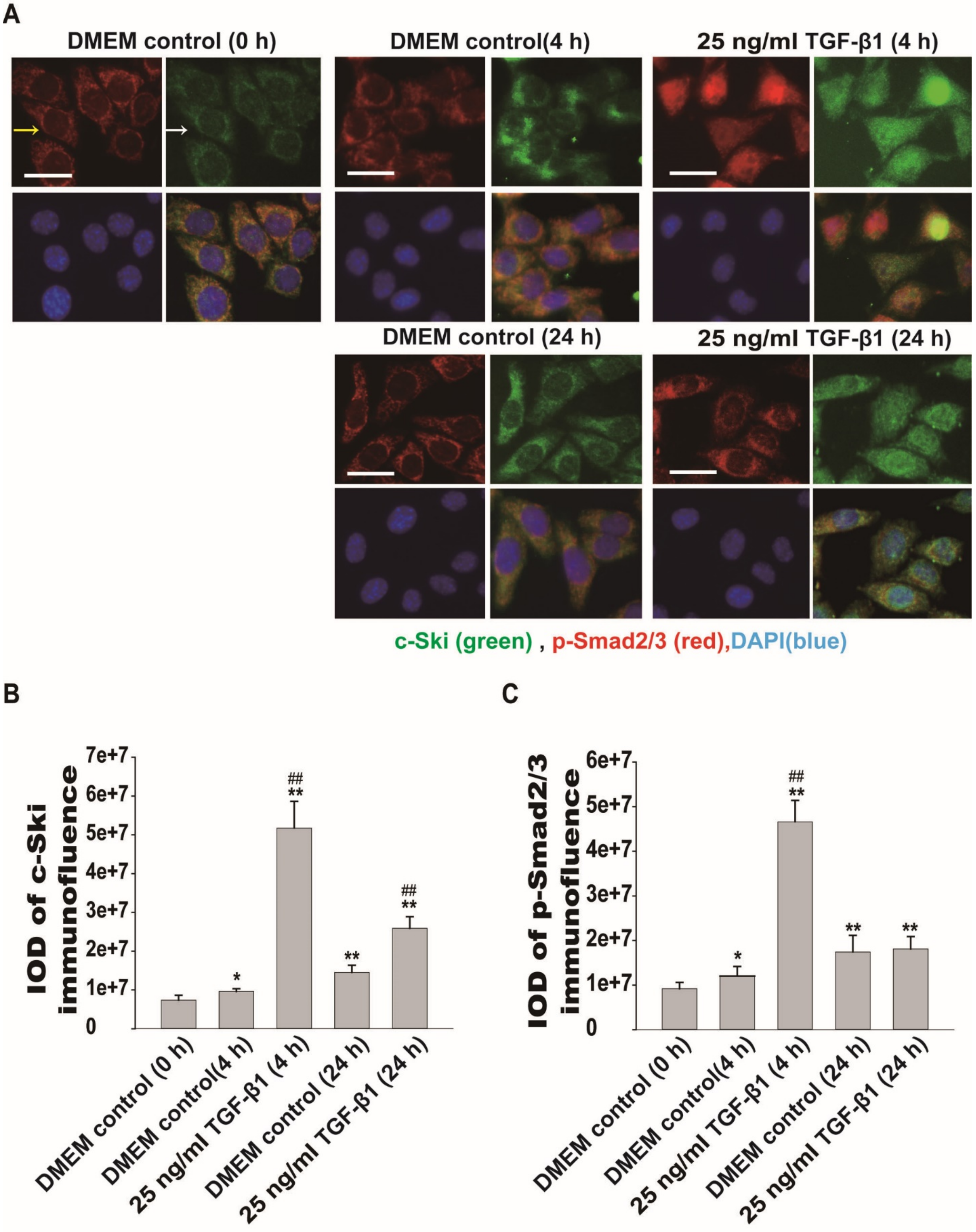
A
DMEM control (0 h)
DMEM control(4 h)
25 ng/ml TGF-β1 (4 h)
DMEM control (24 h)
25 ng/ml TGF-β1 (24 h)
c-Ski (green) , p-Smad2/3 (red),DAPI(blue)
B
IOD of c-Ski immunofluence
0
1e+7
2e+7
3e+7
4e+7
5e+7
6e+7
7e+7
*
##
**
**
##
**
DMEM control (0 h)
DMEM control(4 h)
25 ng/ml TGF-β1 (4 h)
DMEM control (24 h)
25 ng/ml TGF-β1 (24 h)
C
IOD of p-Smad2/3 immunofluence
0
1e+7
2e+7
3e+7
4e+7
5e+7
6e+7
*
##
**
**
**
DMEM control (0 h)
DMEM control(4 h)
25 ng/ml TGF-β1 (4 h)
DMEM control (24 h)
25 ng/ml TGF-β1 (24 h)

Fig. S3

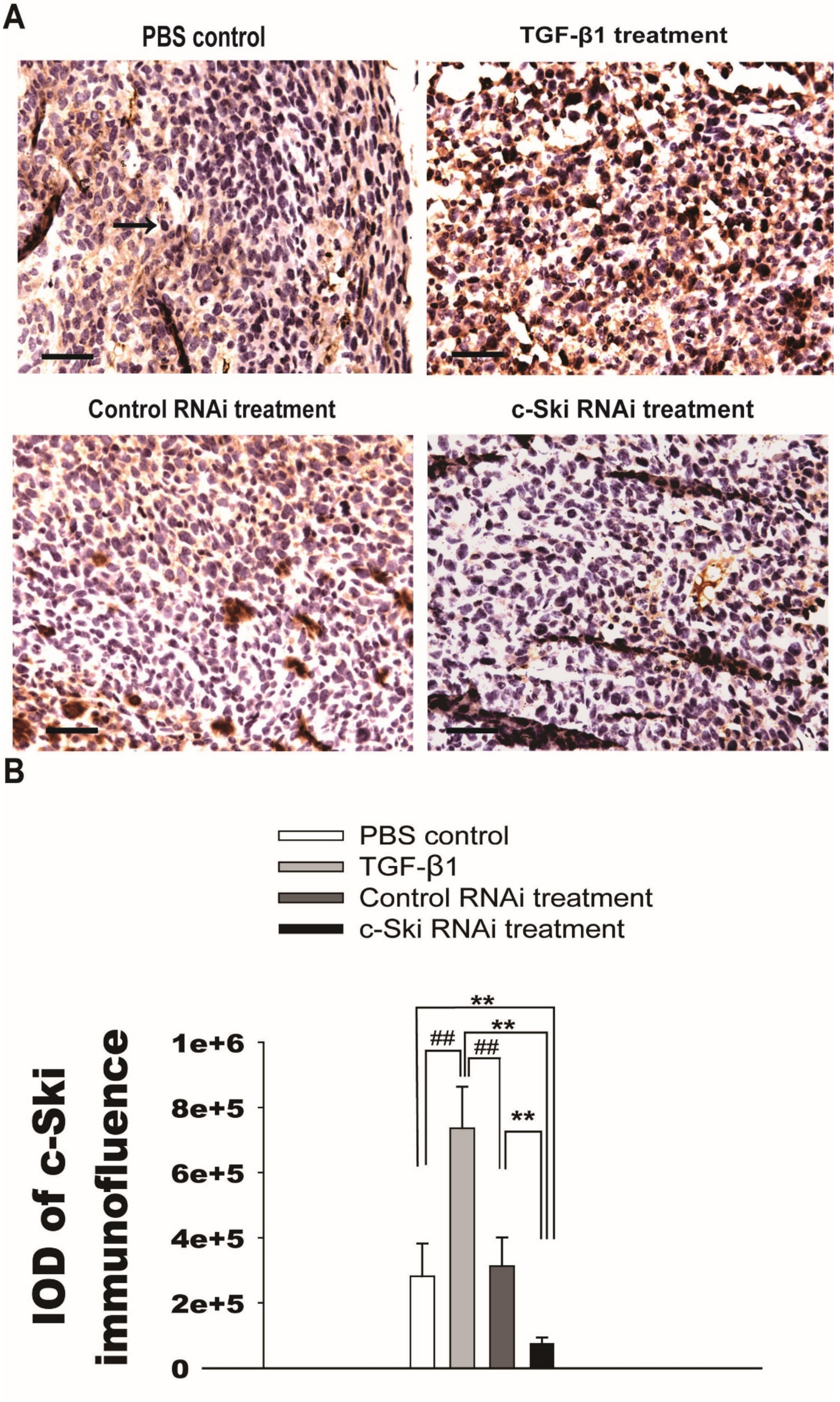
A
PBS control
TGF-β1 treatment
Control RNAi treatment
c-Ski RNAi treatment
B
PBS control
TGF-β1
Control RNAi treatment
c-Ski RNAi treatment
IOD of c-Ski immunofluence
1e+6
8e+5
6e+5
4e+5
2e+5
0
**
##
**
##
**

Table SI Summary of the clinicopathological variables for the dermatofibrosarcoma protuberans patients

Cases	Sex	Age	Site	Primary/recurrent	Tumor differentiation	The Ski expression
1	male	39	buttock	P	low	2+
2	female	50	thigh	R	high	1+
3	male	29	Shoulder	P	low	1+
4	male	50	chest	R	Moderate	2+
5	female	65	neck	R	low	2+
6	female	23	chest	R	high	1+
7	male	50	abdomen	P	high	-
8	male	77	back	R	low	1+
9	female	43	abdomen	R	Moderate	2+
10	female	38	waist	P	high	1+
11	male	12	back	P	low	2+
12	male	43	head	P	high	-
13	female	58	back	P	Moderate	1+
14	male	27	abdomen	R	low	1+
15	male	38	Shoulder	P	low	1+
16	female	22	abdomen	R	Moderate	1+
17	male	53	abdomen	R	Moderate	3+

**18**	female	37	abdomen	P	low	**1+**
**19**	male	36	neck	R	low	**2+**
**20**	male	43	abdomen	R	high	**1+**
**21**	female	27	arm	R	Moderate	**1+**
**22**	male	26	leg	P	low	**3+**
**23**	female	61	chest	R	low	**3+**
**24**	male	59	abdomen	P	Moderate	**2+**
**25**	male	70	buttock	P	low	**2+**

Table SII Multivariate logistic regression analysis

**Model Summary**			
Cox＆Snell square		Nagelkerke square	McFadden square
0.489		0.557	0.319
**Likelihood Ratio Test**			
	chi square	Df	p
Age	0.050	2	0.975
Sex	2.117	2	0.347
Primary or Recurrence	1.796	2	0.407
The expression level of Ski	13.433	2	0.001
**Variables in the equation**			
a	Wald	Df	p
Poor differentiation			
Age	0.025	1	0.874
Sex	1.622	1	0.254
Primary or Recurrence	1.558	1	0.261
The expression level of Ski	611.931	1	0
Moderate differentiation			
Age	0.047	1	0.823
Sex	1.668	1	0.693
Primary or Recurrence	1.596	1	0.660
The expression level of Ski	-	1	-

Note：a:Reference category(Well differentiation).
